# Quality assessment of protein model-structures based on structural and functional similarities

**DOI:** 10.1186/1471-2105-13-242

**Published:** 2012-09-21

**Authors:** Bogumil M Konopka, Jean-Christophe Nebel, Malgorzata Kotulska

**Affiliations:** 1 Institute of Biomedical Engineering and Instrumentation, Wroclaw University of Technology, Wybrzeze Wyspianskiego 27, 50-370, Wroclaw, Poland; 2 Faculty of Science, Engineering and Computing, Kingston University, Kingston-upon-Thames, KT1 2EE, United Kingdom

## Abstract

**Background:**

Experimental determination of protein 3D structures is expensive, time consuming and sometimes impossible. A gap between number of protein structures deposited in the World Wide Protein Data Bank and the number of sequenced proteins constantly broadens. Computational modeling is deemed to be one of the ways to deal with the problem. Although protein 3D structure prediction is a difficult task, many tools are available. These tools can model it from a sequence or partial structural information, e.g. contact maps. Consequently, biologists have the ability to generate automatically a putative 3D structure model of any protein. However, the main issue becomes evaluation of the model quality, which is one of the most important challenges of structural biology.

**Results:**

GOBA - Gene Ontology-Based Assessment is a novel Protein Model Quality Assessment Program. It estimates the compatibility between a model-structure and its expected function. GOBA is based on the assumption that a high quality model is expected to be structurally similar to proteins functionally similar to the prediction target. Whereas DALI is used to measure structure similarity, protein functional similarity is quantified using standardized and hierarchical description of proteins provided by Gene Ontology combined with Wang's algorithm for calculating semantic similarity. Two approaches are proposed to express the quality of protein model-structures. One is a single model quality assessment method, the other is its modification, which provides a relative measure of model quality. Exhaustive evaluation is performed on data sets of model-structures submitted to the CASP8 and CASP9 contests.

**Conclusions:**

The validation shows that the method is able to discriminate between good and bad model-structures. The best of tested GOBA scores achieved 0.74 and 0.8 as a mean Pearson correlation to the observed quality of models in our CASP8 and CASP9-based validation sets. GOBA also obtained the best result for two targets of CASP8, and one of CASP9, compared to the contest participants. Consequently, GOBA offers a novel single model quality assessment program that addresses the practical needs of biologists. In conjunction with other Model Quality Assessment Programs (MQAPs), it would prove useful for the evaluation of single protein models.

## Background

Knowledge of a protein three dimensional structure facilitates understanding of molecular mechanisms that underlie processes essential for living organisms, such as metabolic and signaling pathways, and immunological responses. It can also be used to control these functionalities through drugs designed in silico. Experimental determination of 3D structures is expensive, time consuming and, in some cases, not yet possible. For example, most membrane protein structures are unknown due to the specific environment of cell membranes, their typically large size and dynamic behavior. If homologous structures are available, computer modeling can be an alternative to experimental techniques. In some cases, partial structural information is available, such as residue-residue contact sites, which can also be applied to full protein reconstruction. If there is no structural data on any similar protein, threading and de novo methods can be applied to obtain coarse approximations of the target. Over 80 and 130 automated servers for 3D structure prediction took part in the two latest editions of the Critical Assessment of Techniques for Protein Structure Prediction (CASP8 and CASP 9, http://predictioncenter.org/). Many of them have web interfaces for public use on-line [1-3]. Consequently, all biologists have the ability to generate automatically a putative 3D structure model of any protein. However, the main issue becomes the evaluation of the model quality.

In order to address this challenge of structural biology, many methods have been proposed. These MQAPs fall within two main categories: consensus and single model approaches. Consensus methods aim at ranking model structures of a given target. The 3D Jury [4] was one of the first methods to implement such an approach. It is a simple tool that ranks candidate model-structures generated by different prediction methods. The approach assumes that high ratio of structural overlap between model subsets indicates the true structure. First, pairwise structural similarity scores between all possible pairs of models are calculated. Then, the final score of a model is calculated as the sum of its pairwise scores normalized by the size of the set of models. A similar approach is implemented in other clustering methods such as ModFOLDclust [5,6] and Pcons [7]. These methods also provide estimates of local accuracy of the models. The produced pairwise superpositions of structures are used to calculate average distances between equivalent residues. The lower is the deviation from the average position in the ensemble, the better is the local accuracy of the model. QMEANclust [8] is a slightly different clustering approach. First, a set of TOP 20 or TOP 10 reference models is selected using a single model quality score – QMEAN [9]. Then all models in the set are compared against those reference models. The global quality score of a model is the median of its acquired scores. The latest evaluation of MQAPs reports that, although consensus methods perform generally well, they are usually unable to extract the best model from a list; their strength seems to be mainly in discriminating between good and bad models [10]. Moreover, they highlight that the usage of consensus methods is quite limited for biologists since they are generally interested in estimating the quality of a single model.

This more challenging task is addressed by methods which are often referred to as “true”, “single model” or “real value” MQAPs. They aim at producing an absolute quality score based on a single model-structure. This class covers a whole spectrum of methods that vary both in terms of computational techniques and general approach to the quality assessment. Although a few methods make use of knowledge and physics based force fields that evaluate atomic interactions [11-13], most approaches are based on statistical potentials. PROVE [14] estimates deviations from standard values of atom and residue volumes that were extracted from high quality PDB structures. Alternatively, Delaunay triangulation is used to identify quadruples of interacting residues [15]. These quadruples are classified into 5 classes. Database structures are used to define a statistical pseudo-potential that is used to calculate the likelihood of observing the defined classes of quadruples in native-like structures. In order to evaluate a model-structure, ConQuass [16] employs a statistical propensity matrix based on evolutionary conservation and surface accessibility profiles of proteins of known structures. Verify3D [17] calculates a structure based profile of the evaluated model that takes into consideration solvent accessible area and polarity of residues. It assigns each residue to one of 18 environmental classes and then verifies the compatibility of the profile and a given sequence, based on residue specific environmental propensities. TUNE [18], similarly to Verify3D, evaluates the compatibility between the structure and sequence. However, in this case 25 structural descriptors are used to define the local environment, then a neural network is employed to predict the probability of finding a given amino acid in a specified environment in native-like proteins. ProQ [19] methods also employ artificial neural networks - these are trained to predict the overall quality of a model based on structural features such as atom-atom, residue-residue interactions, solvent accessibility and secondary structure predicted quality. ProQlocal [20] is an extension of ProQ, which evaluates the local quality of models and also incorporates an additional module that assesses the reliability of a target-template sequence alignment.

The results of assessments of single model methods indicate that although there is some progress, their performance remains relatively poor, in particular when compared to consensus methods [10,21,22]. In CASP9, the best “single model” MQAP was ranked 18-th in the global QA category. Secondly, an important issue is that improvement in the field is currently associated with already existing methods since there were no conceptually new approaches among the best performing groups. In order to address those two issues, in this work we focus on developing a new single model MQAP based on novel principles, involving functionality of a target protein.

It was shown [23] that structural similarity between two proteins is monotonically dependent on their sequence similarity. Moreover, homologus proteins usually perform the same or similar function, e.g. 50% of sequence homology allows for correct function annotation in 94% of examined proteins [24]. On the other hand, derivation of a protein function from the sequence alone may lead to misannotations [25-27]. This is due to the fact that relations between sequence, structure and function of proteins are not straightforward. There are some versatile folds (e.g. TIM-barrel, Rossman fold) that are adopted by proteins which perform completely different functions [26,28,29]. This is also supported by the fact that the number of known unique folds (as defined by SCOP [30]) from Protein Data Bank (PDB) [31] equals 1393 (as of 11.2011), while the number of all non-redundant structures (below 30% of sequence similarity) is 18,132. The ratio leads to the conclusion that individual domains of different proteins can adopt very similar shapes. Conversely, the same molecular functions can be performed by proteins of different folds [26]. Still general studies show that close or identical molecular functions impose specific spatial constraints and induce structural similarity of proteins [32,33]. Thus, proteins that are similar in terms of their function may also be similar in terms of the structure. In the study by Wilson et al. [34] it was shown that protein functional annotation from its structure is easier than that from its sequence - the error rate is below 20%. Moreover, Hvidsten et. al [35] report that purely structure-based function predictions complement sequence-based predictions and correct predictions can also be provided when no sequence similarity exists. These three components - sequence, structure, and function - often carry different and complement information. This complementarity can be utilized in the quality assessment of model-structures.

Despite the fact that there are tools that infer protein function based on available structural data [36], one of the concepts that still has not been extensively explored is use of the functional description of proteins as the input to the structure prediction process. Only a few papers report attempts to apply this relation [37,38], however not using Gene Ontology (GO) [39]. Here we introduce the use of formal description of protein function with GO and its semantics.

In this study we present GOBA - Gene Ontology-Based Assessment -which is a single model MQAP based on the assumption that a high quality protein model-structure is likely to be structurally similar to native proteins whose functions resemble the function of the target protein. Conversely, low quality model-structures should be structurally less correlated with proteins of similar function.

The proposed approach uses the semantic similarity of functional annotations provided by the GO Consortium [39] to quantify the functional similarity of proteins. GO provides a tripartite controlled vocabulary of precisely defined GO terms that allow for unambiguous description of three distinct aspects of genes and proteins. The biological process vocabulary refers to the biological mechanism in which a gene or protein participates. The cellular component vocabulary specifies the place where the gene or protein is active within the cell. Finally, molecular function vocabulary describes the biochemical activity of the characterized item. Since the vocabularies are organized into directed acyclic graphs with edges that specify the relations between parent and child terms, semantic tools can be used to express the similarity between GO terms in a quantitative way [40].

In this paper, we first investigate the relation between structural and functional similarities of proteins. Then we validate the performance of GOBA on three datasets of models submitted to CASP8 & CASP9 contests. Finally, we compare GOBA to the quality assessment methods that participated in these CASPs.

## Results

The concept underlying our method was validated using a representative set of protein native structures, while the accuracy of model quality predictions was tested using model-structures of protein targets issued and assessed in the CASP8 and CASP9 contests (see Methods for a full description of datasets). In addition, comparisons were conducted with all MQAPs that took part in the CASP8 and CASP9 events.

### Functional and structural similarities

**Figure 1:**
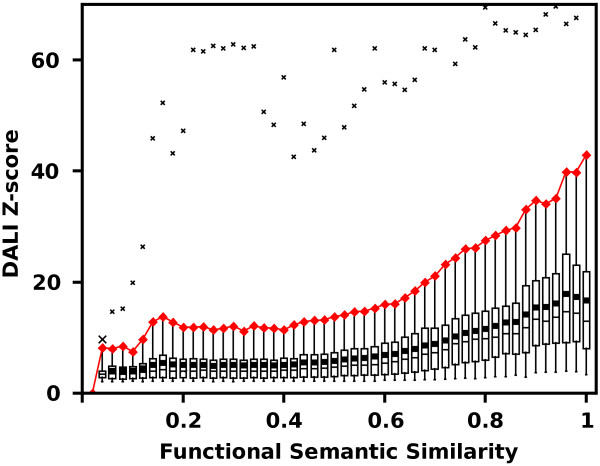
**Dali Z-scores of SNs of a representative set of SCOP structures plotted against their FS values.** The diagram shows in a quantitative way how structure similarity translates to similarity of function. In order to characterize the function-structure relation, all data points were grouped into semantic similarity bins and box-plotted. Box whiskers denote 0.05 and 0.95 quantiles; box edges and the intersecting line are 0.25, 0.75 and 0.5 quantiles respectively; the average values of Z-scores are shown by squares; minimal and maximal values are marked with crosses. The width of each bin was 0.02. The experiment showed that functional and structural similarities are inter-dependent for proteins whose functional similarities are higher than ca. 0.6 . Red line marks the 0.95 quantiles.

Our method is based on the assumption that there is a good correlation between similarity metrics of protein structure and function. This relationship was investigated on a representative, non-redundant set of 5901 native structures from SCOP database [30]. Dali Z-scores of Structural Neighbors (SNs, see Methods) of each protein were plotted against their corresponding Functional Similarity (FS) scores (Figure 1). Over 700,000 protein pairs were compared.

Figure 1 substantiates our hypothesis that the chosen structural and functional similarity metrics are related. Mean and median values of DALI Z-scores in subsequent bins behave monotonically and start to increase significantly once the FS values are greater than ca. 0.6. The plot confirms that in the general case proteins of low FS do not achieve high structural resemblance and high Dali Z-scores impose high mean FS values. However there are exceptions (Figure 1 extremes marked by crosses), which show that structurally similar proteins may in some cases vary in terms of their functions.

**Figure 2:**
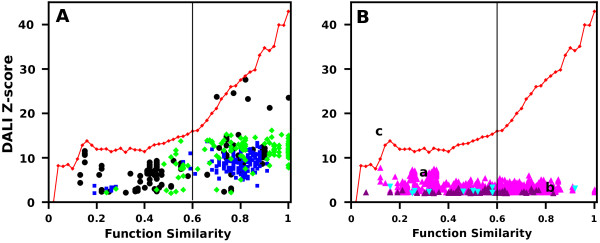
**Dali Z-scores of SNs of exemplary CASP8 model-structures plotted against their FS values.** (**A**) SNs of high quality model-structures (black - T0388TS426_5-D1, GDT-TS = 91.77, green - T0456TS002_1-D1 GDT = 76.44 and blue- T0392TS138_4-D1 GDT = 87.2 ) have a distribution of points similar to that of native proteins SNs (here figuratively indicated by red line, which marks the 0.95 quantiles calculated for SNs of native proteins). (**B**) Neighbors of low quality model-structures accumulate in specific parts of the plot (marked by a, b, c) due to low Z-scores (purple - T0422TS404_5-D1, GDT-TS = 30.00, cyan-T0392TS479_1-D1 GDT-TS = 28.96) or/and low FS values (magenta - T0418TS420_1-D1, GDT = 14.18). The solid vertical line at FS = 0.6 indicates the FS threshold found in the previous experiment with native proteins when function and structure similarity start to depend on each other.

Since high quality (HQ) model-structures should not differ significantly from native proteins, the Z-score vs FS plot of HQ SNs should comply with the same pattern as native proteins. This is illustrated in Figure 2A, presenting three HQ model structures, where the red line denotes 0.95 quantile of native proteins, as in Figure 1.

The FS scores of the most structurally similar SNs of the evaluated HQ model are high, which confirms that such structures are compatible with the molecular function of the target protein. On the other hand, the plot generated for low quality (LQ) model-structures displays a significantly different pattern (Figure 2B). The majority of their SNs show low structural and/or functional similarities and accumulate in three areas (denoted as a,b,c in Figure 2B). Three general cases in LQ model-structures are observed:

a) low FS and low Dali Z-scores, which indicates the prediction process has failed. This is supported by the fact that the produced model is non-natural and unlike any other existing protein;

b) high FS and low DALI Z-scores, which means the model may have certain structural features responsible for the protein function resembling the target function, but the overall similarity is low as it can be seen in cases of convergent evolution.

c) low FS and high DALI Z-scores, which denotes the evaluated model-structure is structurally very similar to another native protein that has a different function from that of the target. This situation should be rare and only occurs when a wrong template structure is chosen for the prediction or in cases of divergent evolution when the global protein structure is conserved, but functions have changed dramatically.

**Figure 3:**
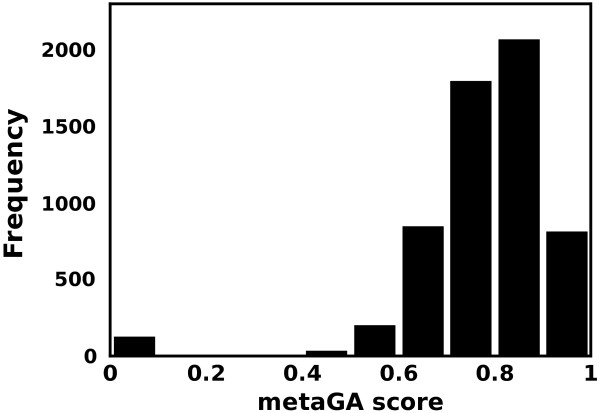
**The distribution of metaGA scores acquired by a representative set of native protein structures which were used as ideal model-structures.** In the case of normalized GOBA metrics, the expected score of an ideal model-structure is 1. Here the average score of analyzed structures is 0.77.

Finally, we complete the evaluation of our assumption by assessing a representative set of native protein structures (5901 structures, for details see Methods) using GOBA scores. As illustrated in Figure 3, which displays the distribution of the metaGA scores assigned to the tested proteins, our method correctly evaluates them as high quality structures: - more than 97% of the native structures under evaluation scored higher than 0.5. In line with the results presented in Figure 1, which highlights the non-linear relationship between structural and functional similarity measures, they do not obtain a ‘perfect’ score of 1, but a relatively high average score around 0.75, whatever GOBA metric was used (Table 1). This can be intuitively explained by the fact that whereas structure is a global property, some functions rely essentially on local functional sites. Therefore, some structural elements may not contain any specific functional information.

**Table 1:** 5901 representative protein structures from SCOP database were scored by the proposed GOBA metrics

	**Mean**	**SD**	**Median**
metaGA	0.77	0.15	0.80
GA_7_	0.76	0.20	0.79
GA_468_	0.74	0.17	0.76
GA_567_	0.73	0.19	0.75
GA_678_	0.76	0.18	0.79
GA_579_	0.77	0.17	0.80
GA_3579_	0.75	0.16	0.77

A small fraction of structures, i.e. 127 out of 5901 (2.15%), did not have enough significant SNs, thus their score was 0 for all the measures (for procedure see Methods section). This shows that our method should not be used for proteins that do not show any structural similarity to already known structures. However, this does not mean that GOBA cannot be used for evaluation of New Fold predictions, which still may display some similarities in those parts of the structure which are responsible for protein function, like active or binding sites.

As our experiments have shown, the relation between metrics used to express protein functional and structural similarity follows a characteristic non-linear pattern, which can be used to distinguish between protein model-structures of low and high quality. Consequently, we believe the assumption behind our approach is valid.

### GOBA validation results

GOBA was validated using three sets of model-structures submitted to CASP contests (see Methods section). The first set comprises models associated to 71 suitable targets offered by CASP8 contest. The other two are sets of models of 16 and 69 targets from CASP9. Validation on the 16 target CASP9 set was a test carried out during the contest (in the text it will be referred to as CASP9-in-contest), while the 69 target set, which includes the CASP9-in-contest targets, was used in a post-contest validation, when more functional annotations became available (the second set is referred to as CASP9). In each validation set the performance was evaluated by calculating “overall” and “per target” Pearson correlation coefficients between observed model-structure quality and for each of the proposed GOBA scores (for details see Methods). Moreover, we tested the ability to identify best quality models by investigating GDT differences (ΔGDT) between GOBA best and objectively best model-structures.

**Figure 4:**
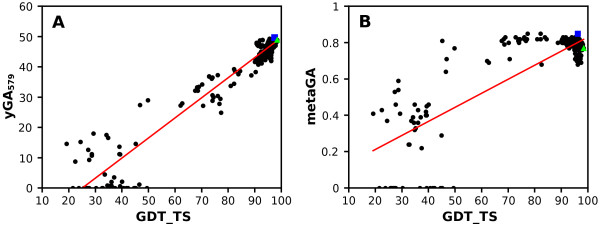
**Quality assessment of T0458 (3dex). Exemplary performance.** GOBA predicted quality scores of an exemplary CASP8 target, yGA_579_(**A**), metaGA (**B**), are plotted against the observed quality of models using the GDT_TS score. Pearson correlations are (**A**) 0.97 and (**B**) 0.84 respectively. Best models according to GOBA metrics are marked with blue rectangles, while the objectively best model is marked with green triangles. The GDT differences between GOBA and objectively best model are 0.98 (**A**) and 2.28 (**B**).

Figure 4 illustrates an exemplary performance of two GOBA metrics chosen as representatives for GA and yGA family of scores, i.e. metaGA and yGA_579_. Results for models of a CASP target (CASP8 T0458) and their correlations with GDT-TS values are shown here. The yGA_579_ score performed better, as illustrated by the correlation value, i.e. 0.97, the linearity of the curve and the ΔGDT (0.98) (Figure 4A). Although the “per target” Pearson correlation coefficient calculated for metaGA is quite high, around 0.84, the GDT-TS/metaGA correlation shows some linearity only for lower quality model-structures, i.e. GDT-TS < 50 (Figure 4 B). Here, scores appear to be independent from GDT-TS for models of better quality, i.e. GDT-TS > 50. Consequently, in this case (Figure 4B), ΔGDT was worse, i.e. 2.28, than in Figure 4A.

**Figure 5:**
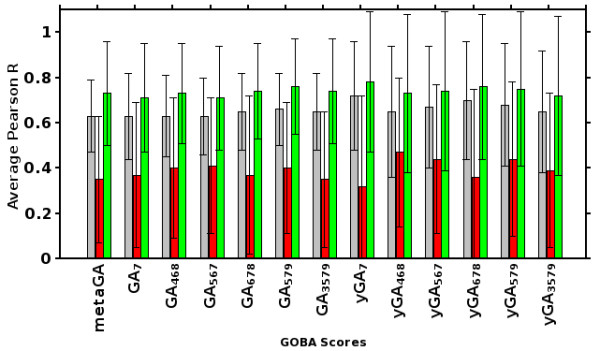
**Comparison of proposed GOBA scores by average per target Pearson R.** Bars show correlations calculated for the CASP8 (gray), CASP9-in-contest (red) and CASP9 (green) sets respectively.

We proposed and validated a number of GOBA metrics. The comparison of their performance evaluated with average per target correlations is presented in Figure 5. Within each validation set the differences in performance of metrics that belong to the same family are small. All GA-family metrics show an approximate correlation of 0.6 and 0.7 in CASP8 and CASP9 sets, respectively. The yGA metrics performed better, with approximate correlations of around 0.7 and 0.75 (see Additional file 1,  2 and 3 for exact values). A significant drop in performance between CASP9-in-contest and the two remaining sets can be noticed for all metrics – this is analysed in details later in this section, however still yGA metrics performed better than GAs. Table 2 provides quantitative results regarding performance for all prediction targets, all validation sets, and selected metrics, i.e. GA_468_, GA_579_, yGA_468_, yGA_579_ and metaGA.

**Table 2:** Pearson correlation coefficients between GOBA and GDT_TS scores of evaluated model structures, calculated in the “over-all” and “per target” evaluations

		**metaGA**	**GA_468**	**GA_579**	**yGA_468**	**yGA_579**
CASP 8	Overall	0.58	0.58	0.61	-	-
	Per target	0.63 ± 0.16	0.63 ± 0.18	0.66 ± 0.16	0.65 ± 0.29	0.68 ± 0.27
CASP9 -in-contest	Overall	0.36	0.49	0.39	-	-
	Per target	0.35 ± 0.28	0.40 ± 0.31	0.40 ± 0.29	0.47 ± 0.33	0.44 ± 0.34
CASP 9	Overall	0.68	0.68	0.7	-	-
	Per target	0.73 ± 0.23	0.73 ± 0.22	0.76 ± 0.21	0.73 ± 0.35	0.75 ± 0.34

These scores were chosen for more detailed analysis, because their FS thresholds span the whole range of possible FS values, which is not the case for those that were omitted. Comparison between the quality of models of different targets cannot be performed using yGA-family metrics since they are a relative single model MQAP, which only compares models of the same target. Consequently, “overall” correlations were only calculated for GA-family metrics.

**Figure 6:**
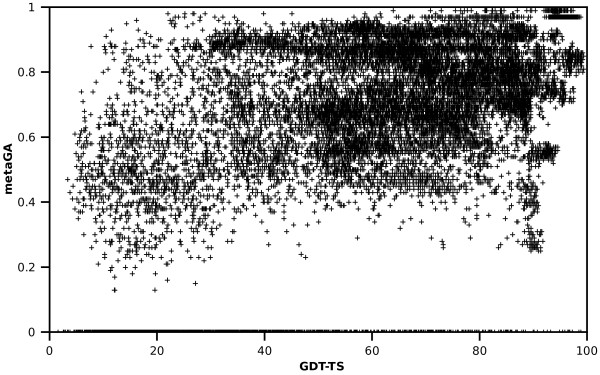
**The correlation of GOBA metaGA score and GDT-TS for all 39,894 CASP8 evaluated models.** A significant number of model-structures scored AUC = 0 since DALI was unable to find more than 5 significant (Z-score >2 ) structural neighbors.

The “overall” correlation analysis in CASP8 set shows that, although GOBA scores are equivocal in respect to GDT-TS values, most model structures that have GDT-TS greater than 50 are assigned scores greater than 0.5 (Figure 6), i.e. 94%, 86% and 91% for metaGA, GA_468_, and GA_579_, respectively. Moreover, values below 0.5 of the respective metrics acquire are associated to 52%, 59%, and 66% of structures with GDT-TS lower than 50. If very poor models are considered, i.e. GDT < 30, the ratios of proteins with scores below 0.5 are 75%, 81% and 85%, for metaGA, GA_468_, and GA_579_, respectively. Similar ratios were acquired for both CASP9 sets (data not shown). This proves that GA metrics are able to discriminate between good and bad models. The calculated "overall" and “per target” correlations for the three "single model" MQAP measures reveal significant correlation with GDT-TS (Table 2). Since the computational cost of GA_468_ and GA_579_ is significantly lower than that of metaGA (see Methods), we would suggest that these approaches are marginally better "single model" MQAPs within the GOBA framework.

**Figure 7:**
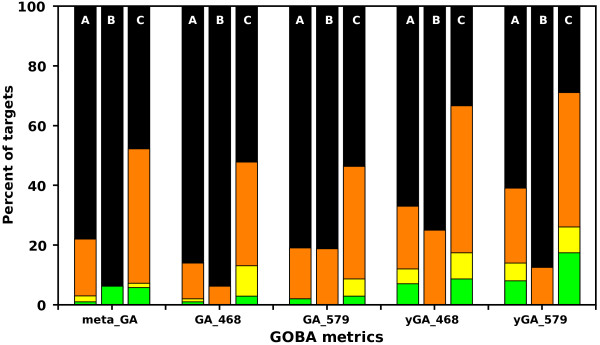
**Analysis of ΔGDT values for selected GOBA measures in CASP8, CASP9-in-contest and CASP9 validation sets.** The bars show in how many cases in a validation set the best selected model was within a certain GDT range from the objectively best model. Sets are labelled with A - CASP8, B - CASP9-in-contest and C – CASP9). Green denotes percentage of targets within 1 GDT, yellow [1,2), orange [2,10), black greater than 10. The yGA-family measures considerably outperform GA metrics.

Whereas metaGA, GA_468_ and GA_579_ results are quite similar, in general yGA family scores performed better, as shown by correlations obtained in the “per target” experiment (Table 2). Also the analyzed ΔGDT values (Figure 7) are in favor of yGA scores. For instance, in CASP9 validation, for 70% of targets their best model-structure indicated by yGA_579_ were within 10 GDT from the objectively best models, and in 26% of cases within 2 GDTs. For metaGA the ratios were 50% and 7% for 10 and 2 GDT thresholds, respectively. GOBA clearly benefits from incorporation of quantitative information from DALI Z-scores. The drawback of using Z-scores is that they cannot be normalized between different protein targets. Therefore, yGA scores are relative: they can only be used for ranking a set of model-structures, no matter how numerous the set is.

The performance of GOBA in CASP9-in-contest test was not as good as in CASP8 and CASP9 validation sets (Table 2, Figures 5, 7). One of the reasons could be that some of the function annotations were derived from homology based annotation with a threshold of 50% of sequence identity (see Methods). However, our post-contest examination of those annotations showed that all of them were correct. The difficulty level of particular targets can differ significantly across CASP targets [41]. CASP9 assessors clearly stated the overall difficulty level of targets in CASP9 was much higher than in the CASP8 and CASP7 editions [10]. The average GDT_TS values calculated for models in our validation sets confirm this fact. Whereas in our CASP8 the average quality of models (measured with GDT-TS) was 60.67, it was much lower in the CASP9-in-contest set, i.e. 44.16. In our CASP9 set it was 51.52. In addition, the fact that Free Modeling targets made for 25% of the CASP9-in-contest set supports the claim that it was much harder than the two remaining sets. There is also a difference in the way models were chosen for the validation sets. For CASP8 set we used as many models as possible, thus models submitted by both server and human groups were downloaded. Since we actively participated in CASP9 QA category, the only models available for the validation set were server-generated models. In order to make the comparison of performance between CASP9-in-contest and CASP9 easier, in the latter set we also used server-generated models only.

We recalculated the correlations in CASP8 set separately for human and server-submitted models (Table 3). The origin of models did in fact influence the performance of GOBA. The results for human models were significantly better.

**Table 3:** Performance of selected GOBA metrics (measured with “per target” correlation) on server-submitted and human-submitted CASP8 models

**Metric**	**Server**	**Human**
metaGA	0.60 ± 0.17	0.75 ± 0.21
GA_468	0.61 ± 0.19	0.73 ± 0.23
GA_579	0.63 ± 0.17	0.77 ± 0.27
yGA_468	0.63 ± 0.3	0.73 ± 0.31
yGA_579	0.66 ± 0.28	0.76 ± 0.28

Although GOBA worked reasonably well for most of the selected targets in CASP-in-contest, its poor performance in case of three targets, i.e. T0604, T0628 and T0630, had a strong negative impact on the average results (Table 4). We investigated those worst cases and found different reasons for GOBA low performance. It was already mentioned in the Assumption Evaluation paragraph that DALI, used by GOBA, was unable to find any SNs for some of analyzed native structures. T0630 was an example of such cases. The lack of similar structures in the database makes it impossible to correctly assess models, since there are no correct templates to which the models could be compared.

**Table 4:** Pearson correlation coefficients calculated for selected GOBA metrics for the CASP9-in-contest test set

	**metaGA**	**GA_468**	**GA_579**	**yGA_468**	**yGA_579**	**yGA_579†**
**T0520**	0.47	0.74	0.38	0.8	0.84	0.98
**T0529 FM**	0.17	0.22	0.14	0.39	0.19	0.10
**T0543**	0.43	0.51	0.61	0.39	0.24	0.96
**T0547 FM**	0.01	−0.01	0.11	0.76	0.76	0.81
**T0561 FM**	0.26	0.37	0.36	0.35	0.38	0.53
**T0580**	0.54	0.64	0.56	0.65	0.57	0.96
**T0584**	0.74	0.76	0.75	0.91	0.92	0.96
**T0596**	0.38	0.43	0.47	0.65	0.66	0.83
**T0600**	0.67	0.68	0.68	0.65	0.64	0.84
T0604* FM	0.33	0.32	0.34	0.81	0.67	0.88
**T0614**	0.47	0.46	0.56	0.59	0.57	0.56
**T0619**	0.17	0.1	0.12	0.21	0.21	0.94
**T0622**	0.57	0.62	0.64	0.49	0.51	0.66
T0628*	0.29	0.38	0.38	−0.14	−0.15	0.36
T0630*	−0.40	−0.4	−0.38	−0.27	−0.26	−0.16
**T0643**	0.58	0.6	0.62	0.25	0.22	0.48

Asterisks mark targets, for which GOBA the performance is explained in text. Last column (marked with a cross sign) lists the results from CASP9 validation for corresponding targets. Scores with the greatest improvements in terms of yGA_579_ were underlined.

**Figure 8:**
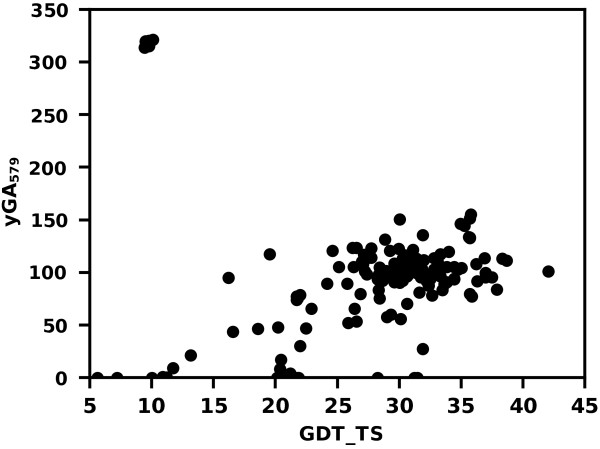
**The performance of yGA**_**579 **_**in assessment of the quality of T0628 predictions.** The method has correctly evaluated most of the predictions, however it failed to assess the quality of models based on a wrongly chosen template. Although the template plays the same physiological role as the target it has a totally different structure.

GOBA quality predictions in the case of T0628 were very good with the exception of 10 structures (Figure 8). These structures were built based on a template protein (information given in the model file) that has a phosphokinase activity similar to the T0628 target, whilst having a totally different structure. That does not comply with the assumption, which underlies our method, that proteins of similar function share common structural features. The 10 model-structures had completely different structural neighbors than the remaining models, thus they should not be compared to the whole ensemble based on yGA scores.

**Figure 9:**
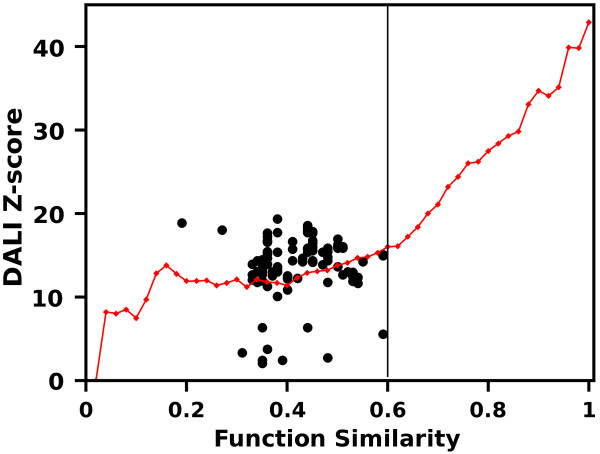
**Dali Z-scores of SNs of T0604 CASP9 target plotted against their FS values.** No correlation between structural and functional similarity of T0604 native structure SNs can be observed; this is the reason for weak performance of GOBA in this case. The red line marks the Z-score 0.95 quantiles calculated for SNs of a representative set of native proteins. The SNs of T0604 violate the pattern acquired from the analysis of native structures.

Finally, a weak correlation between functional and structural similarity in case of SNs of T0604 was the reason of a poor performance in assessing T0604 model-structures (Figure 9). Although many proteins were structurally similar to the target, they displayed low functional similarity. In this case even high quality model-structures, with high structural similarity to T0604 SNs, would not be able to acquire high GOBA scores.

The method performed much better in the post contest validation (Table 2, Figure 7) with new structural and annotation data available. The last column in Table 4 allows to compare “per target” correlations acquired for yGA_579_ in CASP9-in-contest to corresponding values in CASP9. A general improvement of all the results can be noticed; however three targets contributed most to the increase of the correlation average: T0543, T0619 and T0628. Processing of those targets was examined in details. The reason for such significant improvement of correlations was that new structural neighbors of model-structures could be found in the PDB database, which allowed for a more precise model evaluation. For each evaluated model we calculated the median value of Z-scores of its SNs and averaged those medians over all models within each of T0543, T0619 and T0628. In case of all three targets, the average values were significantly higher for results from CASP9 set than from CASP9-in-contest.

### Comparison to state-of-the-art MQAPs

In the last step of the validation process, all the proposed GOBA scores were compared to all MQAPs that participated in the Quality Assessment (QA) category of the CASP8 and CASP9 contests. We retrieved QA predictions from CASP data archive (http://predictioncenter.org/download_area/), and calculated the “per target” Pearson correlations coefficients (similarly as at http://predictioncenter.org/casp8/qa_analysis.cgi) and ΔGDT values for MQAPs that submitted predictions to at least 50% of targets from an appropriate validation set (71 targets in CASP8 or 69 in CASP9).

**Figure 10:**
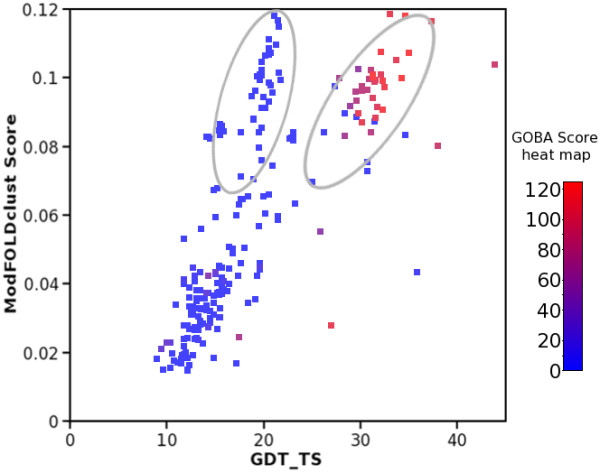
**The example of T0429 presents a case where GOBA is superior over all other methods in CASP8.** Assessment of models submitted by ModFOLDclust. The models formed two structural clusters, which can be observed as two distinct groups (encircled) on the ModFOLDclust vs GDT_TS graph. All points are colored using a blue-red color scale, which is based on GOBA yGA_579_ scores. Blue and red colors are assigned, respectively, to worst and best models in terms of yGA_579_. In this case the consensus method erroneously treated the two clusters as equivalent, while GOBA correctly assigned lowest scores to models from the first cluster and higher scores to models from the second one.

In terms of the average “per target” correlation, yGA_579_ was the best performing GOBA metric. In case of T0429-D1, T0504-D3 CASP8 targets and T0575 CASP9 target, GOBA was the best among all compared methods. In addition, yGA_579_ was also one of the top-performing methods for many others, e.g.T0504-D1, T0501-D1 (CASP8) or T0563 (CASP9) (see Additional file 4 and Additional file 5). The example of T0504 domains shows that our method can perform as well as the best state-of-the-art approaches, whilst the case of T0429-D1 reveals that GOBA can have an advantage over these methods. This is illustrated by the two large clusters formed by the models of T0429 submitted to the CASP8 contest (Figure 10). While clustering methods estimated the quality of models from the two clusters as equal, GOBA could distinguish between them and provide better quality estimates.

**Figure 11:**
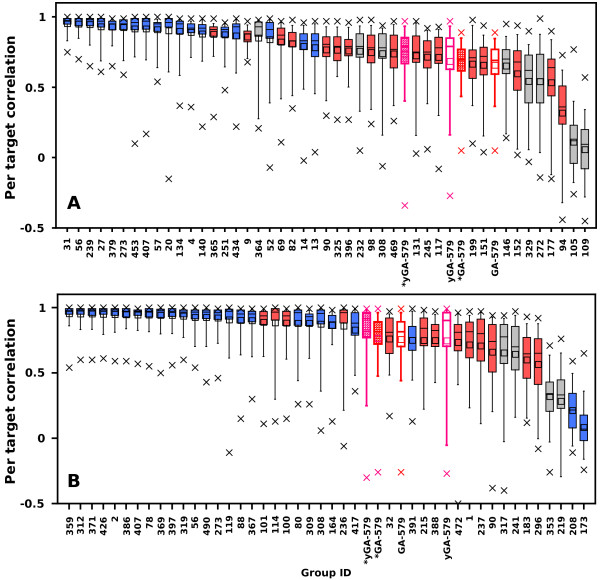
**Performance comparison between GOBA and model quality assessment programs from (A) CASP8 and (B) CASP9 based on per target correlations.** The boxplots show the percentiles of the correlation of particular scores with the GDT-TS per- target; whiskers - 0.05 and 0.95; box edges 0.25 and 0.75; the intersecting line - 0.5; the average values of correlation are shown by squares; minimal and maximal values are marked with crosses. Boxes with pink and red borders show the “relative” and “single model” GOBA scores respectively. The basic version of GOBA scores are denoted by empty boxes, while the striped ones show the performance of GOBA after applying the outlier exclusion procedure. Blue solid boxes are consensus methods, while red ones are single and quasi-single model methods. Gray boxes are methods which could not be classified in either of those two categories.

**Figure 12:**
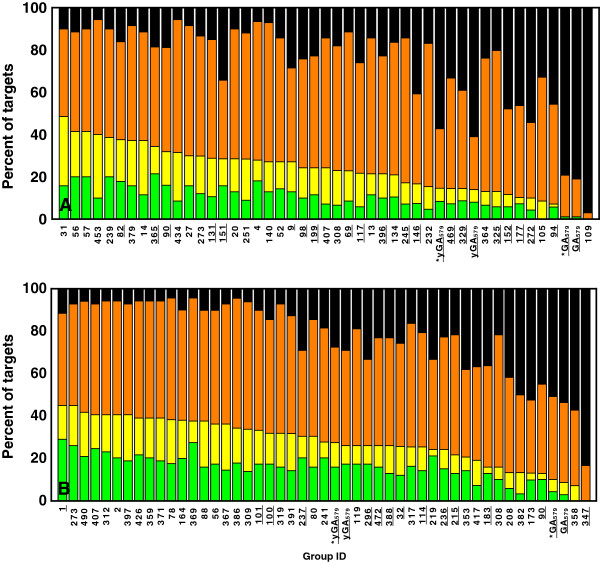
**Performance comparison between GOBA and model quality assessment programs from (A) CASP8 and (B) CASP9 based on ΔGDT analysis.** The bars show the percentage of cases where ΔGDT for a given MQAP was: less than 1 - green , [1,2) - yellow, [2,10) – orange or greater than 10 - black. Single and quasi-single model methods are underlined. Asterix next to GOBA score name denotes that outliers exclusion procedure was applied.

Based on average “per target” correlations and ΔGDT values we constructed rankings of CASP8 and CASP9 participants extended with the best performing GOBA scores (one from yGA-family and one from GA-family), i.e. yGA_579_ and GA_579_ (Figures 11 and 12). Our best scoring scheme - yGA_579_ with the exclusion procedure modification (see next paragraph) was ranked 31-st in CASP8 (Figure 11a) and 27-th in CASP9 (Figure 11b) (based on average per target correlation, pink striped boxes), which fits around the middle of both classifications. It was ranked 10-th and 5-th, if only “single model” MQAPs were taken into consideration. Many MQAPs showed very high correlations with the GDT-TS and differences between the scores of the top-performing methods were not statistically significant, as shown by CASP assessors [21]. The GA-family metrics performed worse than proposed relative yGA-family scores, especially in terms of ΔGDT. In the ΔGDT rankings, our best scoring scheme was ranked 34-th in CASP8 (Figure 12a) and 26-th in CASP9 (Figure 12b). It has to be stressed that all GOBA methods in the CASP8 and CASP9 validation sets produced Pearson correlations higher than 0.5 in both “over-all” and “per target” evaluations. This shows that this is a meaningful and valuable approach for the assessment of protein model structures. In addition, GOBA captures information which is not retained by other methods. Since one of the ways to enhance the performance of MQAPs is to combine approaches [7], GOBA, which introduces additional source of information, and selected GOBA metrics GA_468 ,_ GA_579 ,_ yGA_468_ and yGA_579_ in particular, are suited to complement more traditional approaches.

### Exclusion procedure modification - results

**Figure 13:**
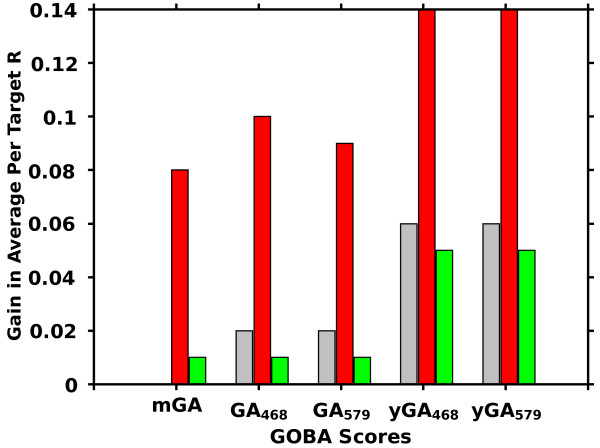
**Gain in “per target” correlations after introducing the outlier exclusion procedure.** The procedure improved the performance of GOBA methods in all sets: CASP8 (gray), CASP9-in-contest (red) and CASP9 (green). The application of the procedure was most beneficial in the CASP9 set.

After CASP9-in-contest test an exclusion procedure was added as a post-processing step of GOBA (for details see Methods). The extended version of GOBA scores, i.e. yGA-family scores, are based on explicit values of Z-scores, which are not absolute measures of structural similarity. Therefore yGA scores should only be used to assess models that share a common structural neighborhood. In some cases of CASP targets this condition was not fulfilled: there were models so highly different that their associated SNs generated by DALI were unrelated. It resulted in exceptionally high differences in scores acquired by models with different structural neighborhoods (e.g. the models of T0628 from CASP9 that were mentioned). The exclusion procedure benefits from a “majority voting” approach. It excludes suspicious and outlier models from the evaluation (for example, the 10 models mentioned in the T0628 case) based on too high yGA-family scores. It was shown that the procedure improved the performance of GOBA quite significantly (Figure 13, Table 5). In some cases, however, it may exclude HQ model structures at a relatively poor background – majority of the ensemble is low quality models. Therefore each excluded model should be manually examined. Since this procedure was not tested during CASP9 contest, it is treated here as an addition to the basic methods.

**Table 5:** Performance of GOBA metrics (measured with “per target” correlation), after additional step of processing – exclusion procedure

	**metaGA**	**GA_468**	**GA_579**	**yGA_468**	**yGA_579**
CASP 8	0.63 ± 0.17	0.65 ± 0.16	0.68 ± 0.14	0.71 ± 0.22	0.74 ± 0.20
CASP9 -in-contest	0.44 ± 0.20	0.50 ± 0.23	0.48 ± 0.21	0.61 ± 0.20	0.58 ± 0.20
CASP9	0.74 ± 0.22	0.74 ± 0.22	0.76 ± 0.21	0.78 ± 0.29	0.80 ± 0.28

### The influence of GO terms specificity on GOBA performance

Since Gene Ontology is a hierarchical structure, the GO terms, which are associated to proteins, can have different levels of specificity, i.e. depth in GO graph. We have defined the specificity of an annotation set as the maximal depth of a GO term annotated to a protein. The analysis did not show any significant correlations (Additional file 6). This means that even an approximate knowledge of the function of a protein is sufficient to use GOBA.

## Conclusions

We presented GOBA, a MQAP that estimates quality of single protein model-structures. It is a novel approach, which evaluates the compatibility between the structure of a model and its expected function. Here DALI is used to measure structure similarity between proteins. Protein functional similarity is quantified using the Gene Ontology and semantic similarity of GO terms.

The single model approach was additionally extended to a method that allows ranking models. An important advantage of the extension is that the size of the evaluated model-structure set is not important. This is not the case with available consensus MQAPs, whose accuracy may be limited if the set of candidate models is small and heterogeneous in terms of structure. We reported on 5 selected GOBA metrics, i.e. GA_468 ,_ GA_579 ,_ yGA_468 ,_yGA_579_ and metaGA. After validation of the correlation between the functional and structural similarity metrics on actual protein structures, the proposed quality measures were exhaustively evaluated on model-structures submitted to the CASP8 and CASP9 contests. The yGA_579_ score showed the best performance in selecting the optimal model-structures. It fits among the best CASP8 and CASP9 QA single model participants. The quality estimates of examined models provided by the best GOBA measure, yGA_579_, showed Pearson correlation of 0.74 and 0.80 to the observed quality of the models. It also achieved the best correlation for T0429-D1, T0504 -D3 (CASP8), and T0575 (CASP9) targets, when compared to other CASP participants.

As a consequence, GOBA offers a novel MQAP that addresses practical needs of biologists. In conjunction with other MQAPs, it would prove very useful for the evaluation of single protein models. In addition, we demonstrated that GOBA is beneficial in situations where clustering methods encounter problems assessing model-structures, e.g. due to multiple, equally large structure clusters. Therefore, the proposed approach, as a novel method, could also successfully contribute to a consensus method for ranking protein model-structures.

## Methods

### Assumption evaluation set

The basic assumption that underlays the method was tested on a representative set of SCOP non-redundant structures (30% sequence similarity cut-off was used), which gave 5,901 proteins, for which GO functional annotations were found. Structural Neighbors of those native structures were retrieved from DALI database server (http://ekhidna.biocenter.helsinki.fi/dali/start as of 05.2012). DALI Z-scores of their 733,517 SNs were plotted against their corresponding FS scores (Figure 1).

### CASP8 and CASP9 validation sets

In the study we used three validation sets of models. We refer to them as CASP8, CASP9-in-contest and CASP9 sets.

In total, CASP8 issued 121 prediction targets. Among them, 71 had both suitable molecular function annotations, i.e. GO terms, and structural neighbors. Since some of these targets were divided by CASP assessors into domains, our final CASP8 validation set consisted of 100 target domains (their full list is provided in Additional file 7). Two of them were acknowledged as particularly difficult targets since CASP classified them as Free Modeling targets, i.e. T0416-D2 and T0513-D2. In total, 39,894 submitted model-structures and their associated assessment headers were downloaded from the CASP8 website (http://predictioncenter.org/download_area/CASP8). Regarding GO annotations we used UniProtKB-GOA (GO Annotation@EBI) annotation database downloaded from http://www.geneontology.org/GO.downloads.annotations.shtml as of 12.2009. The Gene Ontology was downloaded in the OBO v1.2 format from http://www.geneontology.org/ as of 11.2009. Functional Similarities were calculated with our in-house implementation of the Wang algorithm [40].

We also evaluated GOBA by blind test, using selected CASP9 targets as a participant of the CASP9 contest. Officially we submitted model quality predictions produced only by the metaGA (GOBA_Wroc_PL, Gr id 358) and GA_7_ (GOBA_PL_07, Gr id 347) metrics. Other metrics, from yGA and GA families, were not registered at the contest but they were simultaneously evaluated on the same set. Their results are presented in this work. CASP9 offered 116 targets in two categories: “server” and “human/server”. We limited the CASP9-in-contest to “human/server” category. Since our approach relies on reliable function annotation, we could only assess the model-structure quality of targets for which we could estimate GO terms with some confidence, i.e. 16 out of the 60 targets proposed in the human/server category. A significant number of those targets, i.e. 4, belong to the Free Modeling category. The whole CASP9-in-contest validation set comprised of 4,319 structures submitted to the contest by server prediction groups (http://predictioncenter.org/download_area/CASP9). Gene Ontology annotations were acquired according to the procedure given in the next section.

Finally, when more annotation data became available, the CASP9-in-contest test was followed by a full validation study – CASP9. The set comprised 5109 model_1 structures associated to 69 targets (which included targets from CASP9-in-contest set) submitted to CASP9 contest by server groups (http://predictioncenter.org/download_area/CASP9). In CASP9 validation we used functional annotations from UniProtKB-GOA (GO Annotation@EBI) as of 05.2012 and Gene Ontology downloaded in the OBO v1.2 format as of 05.2012. We also used an updated DALI structural database (as of 05.2012, last update reported at the server site 03.2011 http://ekhidna.biocenter.helsinki.fi/dali/start).

### GO term annotation during CASP9 contest

As a pre-requisite we assume that a target molecular function is known. Targets were fed into AmiGO [42], which searches for annotations in the GO database. If a target did not have any associated GO term, homology based annotation was considered. Using the BLAST [43] interface of AmiGO, GO terms of the target were inferred from the GO annotated proteins displaying at least a 50% sequence identity. As a consequence of this procedure, some targets may be associated incorrect GO terms.

### Validation procedure

For evaluating the performance of our method we adopted a procedure similar to that used by the CASP assessors of the QA category [10,21,22]. The Global Distance Test total score (GDT-TS), which is a measure that evaluates the best superposition of a model-structure and the native structure [44], is used to quantify the quality of protein structure predictions. Then Pearson correlation coefficients between GDT-TS and each of the proposed GOBA scores were calculated. In each validation set the correlations were calculated collectively for all downloaded model-structures for all targets, as well as separately for each target. Here we referred to them as “overall” and “per target” correlations.

A metrics ability to choose the best among a set of models was also measured. This was done by calculating the GDT difference between the predicted best model and the objectively best model, i.e. the one with the highest GDT-TS score. Here we referred to the measure as ΔGDT. As in [10], percentage of targets where ΔGDT falls into one of four GDT bins: < 1 , [1,2), [2,10), >10 were analysed.

### Calculation of protein function similarity

GO is used to describe the functional aspect of proteins in a standardized way. A protein can be annotated with a set of GO terms. Following suggested guidelines from [45], Wang's method [40] was selected for calculation of semantic similarity between protein functions. The algorithm allows the evaluation of pairwise similarity of GO terms, and, consequently, can be used to compare sets of terms assigned to different proteins. We define the value produced by the comparison of the function(s) of two proteins, i.e. two GO term sets (taken as provided by the Gene Ontology Annotation - UniProtKB-GOA Database), as the Function semantic Similarity score (FS). Although both, the biological process in which the protein is involved and the localization of the molecule within the cell, influence its structure, their impact is indirect and ambiguous: the fact that two proteins participate in the same metabolic pathway or are active in the same environment does not mean they will share similar structure. Consequently, in this study, only “molecular function” GO terms were used for function comparison. Since the presented method relies on GO terms, its usage is limited to target proteins annotated with the ontology.

### Availability of GO term annotations

We have analysed the general availability of functional annotations in the Uniprot database (release 2011_03) and the PDB (as of 01.03.11). Uniprot comprises 14,423,061 records out of that 9,241,363 records are annotated with any GO terms and 8,154,096 records (56.5% of the whole Uniprot) are associated with at least a single Molecular Function GO term. In PDB over 90.4% of proteins are assigned GO terms and 84% have function annotations.

We also analyzed the evidence codes for annotations. In Uniprot the vast majority of annotations are Electronically Inferred Annotations (IEA), i.e. 99%. This is quite different in the PDB, where only 69% are IEA, whilst the remaining annotations are derived from experiments and literature statements. (For details see Additional File 8).

### Calculation of structural similarity

Structural similarity of proteins is quantified using the DALI application [46]. It provides a ready-to-go platform for pairwise comparisons of protein structures and high-throughput searches of the PDB database for structurally similar proteins – SNs. The method is sequence independent. It finds similar patterns in the three dimensional arrangement of secondary structure elements, in the pair of compared proteins, and quantifies the comparison with a Z-score metric. The larger is the Z-score value, the larger is the probability that the result is relevant.

In this process, the DALI output “pdb90” is used. By limiting structural neighbors to proteins which share at most a 90% pairwise sequence similarity, we reduce risks of possible bias caused by proteins that are overrepresented in the PDB. Therefore, only a single variant of each structural neighbor is taken into account during quality assessment of models.

### Model quality assessment procedure

**Figure 14:**
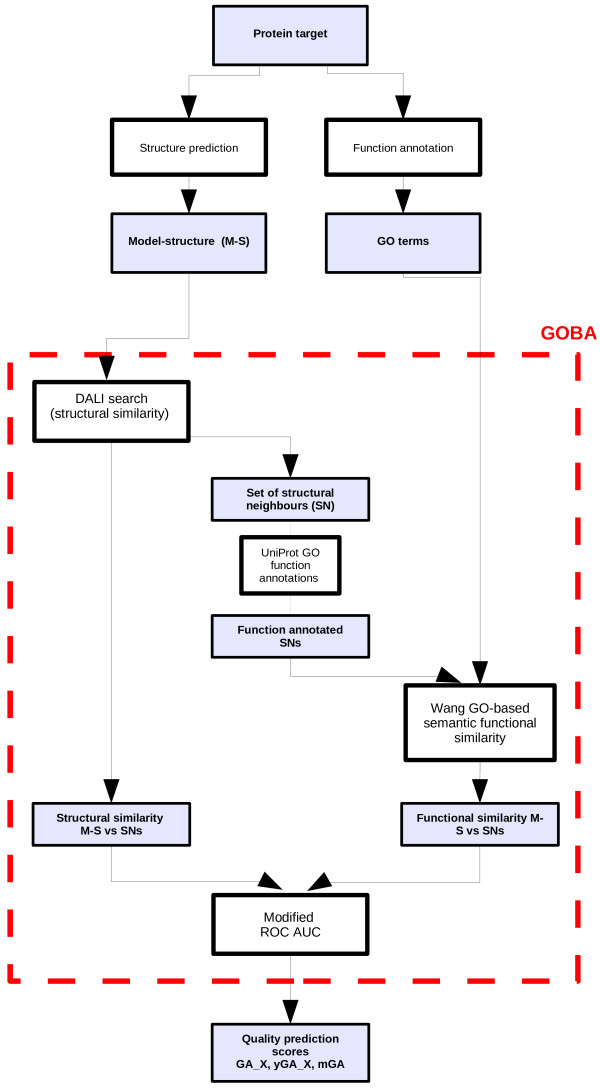
**GOBA work flow.** The scheme shows how data are processed within the proposed approach. The structure of the evaluated model and the GO term annotations of the target are the direct input information. The method produces a number of global quality scores.

The whole work flow of GOBA is depicted in Figure 14. The starting point is the target protein. This protein needs to be annotated with Molecular Function GO terms. For this purpose GO annotations from for example Uniprot can be used. A more demanding alternative is conducting wet lab experiments for the function determination. Putative 3D model-structures can be generated using available structure prediction methods. Next, the functional annotations and the structure are fed into GOBA. The algorithm starts by constructing the Similarity List (Slist) of SNs. First, SNs of the evaluated model-structure are identified using DALI. This is followed by calculations of the FS score between every SN and the target protein. Thus, a single record in the Slist comprises the SN name, the DALI Z-score of the comparison between the SN and the model-structure, and the FS score between the SN and the target protein.

Once the Slist is produced, model-structure quality scores are calculated using variations of the Receiver Operating Characteristic (ROC) methodology [47]. We proposed two diverse approaches to plotting the ROC curve. The first approach, which produces GA-scores, follows the standard procedure of plotting ROC. However the second approach, which produces yGA-scores, introduces a significant modification. In both approaches, the SNs are divided into two sets. If the FS of a structural model is greater than a set FS-threshold, it is classified as a positive hit, otherwise it is a negative hit.

Then, in the basic GA procedure, a sensitivity vs specificity ROC curve is plotted for the Slist, with the DALI Z-score assumed as the cut-off parameter. Practically, the ROC curve is drawn in the following way. The Slist is scanned from top to the bottom. If an SN is classified as a positive then the curve moves up by the value of Δ*y* on the sensitivity axis of the ROC curve plot. Otherwise, i.e. if the SN is negative, the curve is moved on the specificity-axis by the value of Δ*x*. Here Δ*y* and Δ*x* are defined as follows:

(1) $$\Delta y=\frac{1}{\text{TP}},\;\Delta x=\frac{1}{\text{TN}}\text{,}$$

where TP and TN are respectively the total sizes of the positive, and negative SN sets. The basic score, called the GA-score (GOBA Assessment score) is defined as the Area Under the plotted ROC Curve (AUC) (eq 2).

(2) $$\text{GA}\left({\text{FS}}_{\text{threshold}}\right)=\overset{\text{TN}}{\underset{i=1}{\sum }}\left(\Delta x\overset{n\left(i\right)}{\underset{j=1}{\sum }}\Delta y\right)$$

where *n*(*i*) is the number of positive SNs occurring in the Slist before reaching the *i*-th negative SN.

In the modified ROC curve approach the curve is plotted slightly differently. Instead of moving up by *Δy* each time a true hit is encountered in the Slist, *Δy* is multiplied by the Z-score value of the SN. Thus, the score for the second approach is calculated as:

(3) $$\text{yGA}\left({\text{FS}}_{\text{threshold}}\right)=\overset{\text{TN}}{\underset{i=1}{\sum }}\left(\Delta x\overset{n\left(i\right)}{\underset{j=1}{\sum }}\left(\Delta y\cdot {\text{Zscore}}_{j}\right)\right)$$

where *Z-score*_*j*_, is the *Z-score* of *j*-th positive SN.

Both GA and yGA scores depend on the FS-threshold as a parameter. In the course of the study we noticed that linear combinations of scores calculated for a number of FS-thresholds produce better correlations with the observed model quality and are more reliable then single FS-threshold scores. Our choice of FS thresholds, producing single scores contributing to our final scores, was somehow arbitrary, however taking into account our results presented in Figure 1. These joined scores are produced similarly for both score families as shown in eq (4,5).

(4) $$GA_{F}=\frac{1}{N}\underset{fs\varepsilon F}{\sum }GA\left(\frac{fs}{10}\right)$$

where

(5) $$F=\left\{fs_{i}:fs\in \left\{2,3,\ldots ,9\right\}\right\}\text{,}$$

(6) $$N=\text{size}\left(F\right)$$

Here F is a set of integer values which, after dividing by 10, give the thresholds that the scores use. For instance, a score GA_579 is calculated as

(7) $$GA_{579}=\frac{\text{GA}\left(0.5\right)+\text{GA}\left(0.7\right)+\text{GA}\left(0.9\right)}{3}$$

Finally, for GA-family we introduce another score - metaGA (Eq. 6) , which does not rely on any parameter since it is calculated using the average of GAs obtained for all FS thresholds in the range (0,1) with a step of 0.01:

(8) $$\text{metaGA}=\frac{\sum_{i=1}^{100}\text{GA}\left(i\cdot 0.01\right)}{100}$$

where *GA(k)* is the GA score calculated at *FS*-threshold that equals *k*.

GA scores are “single model” MQAPs since they provide an absolute model-structure quality score only based on a single model. On the other hand, the yGA scores rely on Dali Z-score values which are not absolute measures of structural similarity since Z-score values are only defined within a given population of structures. Consequently, yGA based GOBA can only be used for comparing model-structures of a given target. However, contrary to consensus methods, yGA scores can be used with model sets of any size; therefore, it is practical for biologist users.

Since, in GA family metrics, Z-scores are only used to rank structural neighbors, they do not impact on the intercomparability of the results.

GA metrics are by definition normalized (AUC of ROC curves). Their values range between 0 and 1. The scores of the best models should approach 1, whereas, unlike the typical AUC-ROC interpretation, the worst ones are expected to score below 0.5 (instead of 0.5). Indeed, poor quality models may produce Slists that include none or just a few positive SNs. If those SNs are in the bottom of the Slist, the AUC is lower than 0.5.

**Figure 15:**
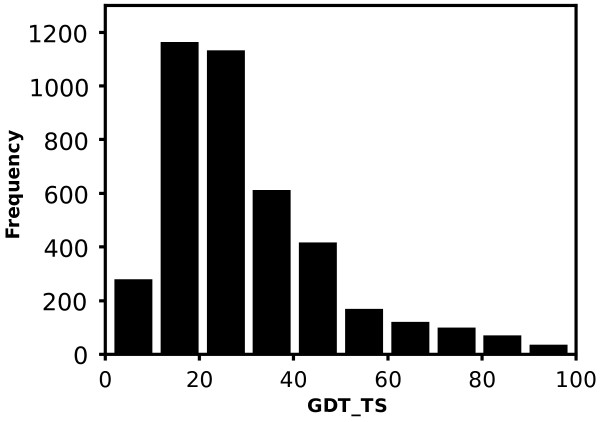
**The distribution of GDT_TS of model-structures with 0 GOBA scores.** If an insufficient number of SNs for a model-structure is found, the quality of the model is assumed to be 0. More than 90% of such models in the study had GDT_TS lower than 50.

If DALI fails to find at least five significant SNs, i.e. Z-score ≥ 2 [48], for an evaluated model, the score of the structure is set to 0.This decision is in line with the assumption that low quality structures do not resemble any real protein. Although this assumption is not always correct, especially when new fold structures do not exhibit structural similarity with known proteins, it proves reasonable - almost 90% of model-structures that were assigned 0 in the validation procedure had a GDT_TS score lower than 50 (Figure 15). Moreover, as structural databases develop, the number of high quality model-structures excluded that way will decrease.

### Outlier exclusion procedure

After CASP9-in-contest test an outlier detection feature was added. Chauvenet's criterion is used to exclude outliers [49]. According to this criterion, a sample is considered an outlier if for a normal distribution of samples, the probability of acquiring a given value is lower than *p =* (2*n*)^-1^, where *n* is the number of samples. Lower and upper cut-off thresholds could be calculated, however since the lower limit of model-structure quality is zero, only the upper one is used. This threshold is calculated as the inverse cumulative distribution function for 1*-p*. All models which exceed the calculated threshold are excluded. In some cases of distributions one may observe a shielding effect, i.e. a sample is not considered as an outlier because there is a more significant outlier. In order to address this “shielding effect”, the exclusion procedure is applied iteratively until all outliers are removed. The removal procedure is the following. First all models are scored according to the standard procedure. Then for each of yGA_468_, yGA_579_, yGA_567_ and yGA_678,_ outlier cut-off thresholds are calculated. A model is excluded if it is an outlier according to any of the scores.

### Availability

GOBA is available for download at http://www.ibp.pwr.wroc.pl/KotulskaLab/materialy/GOBA%20-%20Model%20Quality%20Assessment%20Programe/GOBA_src_BMC_BIO.tgz.

## Abbreviations

QA: Quality Assessment; MQAP: Model Quality Assessment Program; GO: Gene Ontology; SN: Structural neighbors; FS: Function similarity; LQ: Low quality; HQ: High quality; Slist: Similarity list.

## Competing interests

No competing interests declared.

## Authors' contributions

BMK developed the program for model quality assessment, prepared the data sets, carried out the validation analysis and prepared the first draft of the manuscript. JCN proposed the first concept of the method, participated in the design of the study, and participated in writing the manuscript. MK participated in the design of the study, provided supervision over the project, and participated in writing the manuscript. All authors read and approved the final manuscript.

## Supplementary Material

Additional file 1 **CASP8 evaluation.** The file has three worksheets “Overall”, “Per target” and “delta GDT”. The first worksheet provides “overall” correlations of quality predictions produced by all tested GOBA metrics with GDT-TS scores of protein model-structures submitted to CASP8. The second worksheet shows Pearson correlations calculated for model-structures of particular targets. The third worksheet provides the percentages of targets where ΔGDT fell into one of four GDT bins. Click here for file

Additional file 2 **CASP 9–in-contest evaluation.** The content of the file is analogical to the contents of Additional file 1. The file has three worksheets: “Overall”, “Per target” and “delta GDT”. The evaluated models were submitted to CASP9. Click here for file

Additional file 3 **CASP 9 evaluation.** The content of the file is analogical to the contents of Additional file 1 and 2. The file has three worksheets: “Overall, “Per target” and “delta GDT”. The evaluated models were submitted to CASP9. Click here for file

Additional file 4 **comparison of CASP 8 MQAPs.** The file has two worksheets: “Per target” and “Delta GDT”. It shows the performance of selected GOBA measures and the performance of groups that participated in Quality Assessment category of CASP8. The table in “Per target” is sorted by average “Per target” correlation acquired by groups. Average and quartile values along with Minimal and Maximal correlation values are given in the last columns of the table. The second worksheet provides “delta GDT” analysis analogical to those presented in Additional files 1, 2, 3. Click here for file

Additional file 5 **comparison of CASP9 MQAPs.** The file has two worksheets: “Per target” and “Delta GDT”. It shows the performance of selected GOBA measures and the performance of groups that participated in Quality Assessment category of CASP9. The contents of worksheets are analogical to those in Additional file 5. Click here for file

Additional file 6 **dependence of GOBA performance on GO term annotations.** The file shows the correlations between parameters describing protein GO term annotations (number of GO terms, the depth of the most specific GO term, average similarity of GO terms in the set) and GOBA “Per Target” performance in CASP8 and CASP9-in-contest validation sets. Click here for file

Additional file 7 **CASP 8 validation set targets.** The file lists all CASP 8 target domains that were used in our CASP8 validation set. Click here for file

Additional file 8 **GO term availability.** The file has two worksheets, “Annotations” and “Go_evidence”. In the first worksheet, the numbers of GO term annotations, available for protein entries in Uniprot and in PDB, are listed. In the second worksheet the distributions of evidence codes in Uniprot in PDB are presented. The legend for GO evidence codes is also provided. Click here for file
